# Review of the psychometric properties of the Patient Health Questionnaire-9 (PHQ-9) Spanish version in a sample of Puerto Rican workers

**DOI:** 10.3389/fpsyt.2023.1024676

**Published:** 2023-02-14

**Authors:** Ernesto Rosario-Hernández, Lillian V. Rovira-Millán, César Merino-Soto, Marisol Angulo-Ramos

**Affiliations:** ^1^Clinical Psychology Programs, School of Behavioral and Brain Sciences, Ponce Health Sciences University, Ponce, Puerto Rico; ^2^Ponce Research Institute, Ponce Health Sciences University, Ponce, Puerto Rico; ^3^Psychology Program, Social Sciences Department, University of Puerto Rico, Cayey, Puerto Rico; ^4^Instituto de Investigación de Psicología, Universidad de San Martín de Porres, Lima, Peru; ^5^Sociedad Científica Peruana de Enfermería Pediátrica, Lima, Peru

**Keywords:** depression, internal structure, measurement invariance, PHQ-9, random intercept item factor, validity, Puerto Rico

## Abstract

**Background:**

This study aimed to examine the internal structure and assess the psychometric properties of the Patient Health Questionnaire (PHQ-9) in a Puerto Rican sample of workers. This instrument is a nine-item questionnaire, which was conceptualized as a unidimensional structure; however, there are mixed results regarding this internal structure. This measure is used in the occupational health psychology context in organizations in Puerto Rico; nevertheless, there is little evidence of its psychometric properties with samples of workers.

**Materials and methods:**

A total of 955 samples from two different study samples were used in this cross-sectional study design in which the PHQ-9 was used. We conducted confirmatory factor analysis, bifactor analysis, and random intercept item factor analysis to examine the internal structure of the PHQ-9. Moreover, a two-factor model was examined by randomly assigning items to the two factors. Measurement invariance across sex and the relationship with other constructs were examined.

**Results:**

The best-fitted model was the bifactor model followed by the random intercept item factor. The five sets of two-factor models with items randomly assigned obtained acceptable and similar fit indices regardless of the items.

**Conclusion:**

The results suggest that the PHQ-9 appears to be a reliable and valid instrument to measure depression. The more parsimonious interpretation of its scores, for now, is a unidimensional structure. Comparison across sex appears to be useful in occupational health psychology research settings since the results suggest that the PHQ-9 is invariant regarding this variable.

## Introduction

According to the World Health Organization (1), approximately 250 million people worldwide suffer from depression. Meanwhile, the prevalence of depression in Puerto Rico is 18.5% (2). Moreover, depression is the most frequent psychiatric disorder and cause of impairment in the world, ranking second only to diabetes as the primary cause of disability (3).

According to Greenberg et al. (4), 6–7% of full-time US workers suffered from serious depression in the previous year. With employment rates changing by country, employment-related repercussions due to depression are affecting an increasing number of people, both employed and unemployed (5). Furthermore, depression is linked to long-term effects on productivity and is one of the leading causes of workplace absenteeism and presenteeism, in addition to the important social and psychological consequences (6).

According to Grazier (5), despite the fact that multiple studies have confirmed the frequency and prevalence of major depressive disorders in the workforce, considerable gaps in recognizing, screening, treating, and supporting persons with illnesses in workplaces remain. Moreover, depression in working-age individuals results in direct healthcare expenditures as well as indirect costs such as lost working hours, lifetime income loss, and early retirement (6). Loss of productivity is by far the most significant component of the overall economic burden of depression, and it comes at a significant cost to businesses (7, 8). In recent years, statistics in high-income countries have implied that sick days lost due to mental health issues such as depression have increased (9). Mental disorders have trebled their contribution to the cost of permanent disability pensions in Germany, with depression, anxiety, and related neurotic disorders accounting for more than half of these (6).

Meanwhile, some studies [e.g., (5, 10, 11)] argue that the economic impact of workplace depression has become better understood due to more exact quantification of direct and indirect costs. Direct metrics such as absenteeism, disability, and treatment expenditures for the employed can be easily measured using administrative data. On the other hand, Smith et al. (12) indicated that factors that are likely important but difficult to quantify include lost economic opportunity because of depression (e.g., underemployment, missed promotions or overtime, and transferring from full-time to part-time work), and the burden of depression on families or society at a large scale. Most crucially, depressive episodes strike working people early in their jobs and continue to plague them throughout their lives (13).

According to Huarcaya-Victoria et al. (14), it is critical that specialized professionals perform clinical interviews to appropriately diagnose a depressive disorder. Such interviews, however, can be lengthy and are not always appropriate in nonclinical contexts. It is possible to screen and monitor large populations using simple questionnaires, which could improve the detection rates of depressive symptoms in those nonclinical settings.

To assist specialized professionals in identifying at-risk patients, reliable, short, and easy-to-administer depression screening methods are critical (15). Because of its simplicity and excellent psychometric properties, the Patient Health Questionnaire-9 [PHQ-9; (16)] is one of the most used tools for screening depression in primary care settings (17–20). We acknowledge that the PHQ-9 has been translated into several languages and that it is used in several countries in which its psychometric properties have been examined [e.g., (21, 22)]. Nevertheless, we focus our brief literature review on the Spanish version of the PHQ-9 in studies of its psychometric properties conducted in Spanish-speaking countries.

### Brief systematic literature review of the PHQ-9 in Latin America

A brief systematic literature review was conducted to establish the pattern of findings and methodological procedures used in studies of psychometric properties in the general and internal structure of the PHQ-9 Spanish version as recommended by some authors in the literature [e.g., (23)]. The following keywords were used: PHQ-9 AND internal structure OR psychometric properties AND validity AND reliability OR measurement invariance. The review was done through the search engines in the EBSCO, ScienceDirect, Scopus, PubMed, and Google Scholar databases, using “Boolean” connectors between November 2021 and March 2022. Due to the setting of this validation in a Puerto Rican sample, we intended to include studies about the psychometric properties of the PHQ-9 Spanish version. A total of 13 studies that used the Spanish-language PHQ-9 were chosen (see Table 1).

**TABLE 1:** Brief systematic review of the PHQ-9 Spanish version.

#	Study-country	Participants	Factorial design	Factorial loading	Method	Factor relationship	Internal consistency	Measurement invariance
1.	Merz et al. (24) USA	*n* = 479 Latino Women by Language English: *n* = 245 Spanish: *n* = 234	CFA	1-Factor	Estimator: ML (S-B_*S*_)	NR	English Speaking ∝ = 0.84 Spanish Speaking ∝ = 0.85	English-Spanish Speaking (Invariant)
2.	Zhong et al. (25) Perú and USA	*n* = 1,517 Women receiving prenatal care **Age:** 18–48 M(SD): 28.0 (6.2)	EFA and CFA	2-Factor Somatic and Non-Somatic	Estimator: EFA: NR CFA: ML	NR	∝ = 0.81	NR
3.	Arrieta et al. (26) México	*n* = 152 Rural Participants **Sex**: Women 71% **Age**: M (SD): 38 (16)	CFA	1 and 2 Factor	Estimator: WLS	Affe∼∼Som: 0.92	∝ = 0.81	NR
4.	Marcos-Nájera et al. (27) Spain	*n* = 449 Pregnant women **Age:** 19–45 M (SD): 32.9 (5.2)	EFA and CFA	2 and 3 Factors	Estimator: NR	A/C∼∼PS: 0.54 A/C∼∼Som: 0.55 PS∼∼Som: 0.38	∝ = 0.81	NR
5.	Cassiani-Miranda and Scoppetta (28) Colombia	*n* = 441 University Students **Sex**: Female: 63.77% Male: 36.23% **Age:** M (SD) 20.18 (2.59)	CFA	2 Factors	Estimator: ML	Som∼∼Non-Som: 0.91	NR	Gender
6.	Villarreal-Zegarra et al. (29) Perú	*n* = 30,456 **Sex**: Women: 56.7% Male: 43.3% **Age:** 18–98 M (SD): 20.18 (2.59)	CFA	1 and 2 Factors	Estimator: WLSMV	A/C∼∼Som: 0.967 and 0.988	∝ = 0.870 ω = 0.873	Sex, age, education, socioeconomic status, marital status, and residence area
7.	González-Rivera (30) Puerto Rico	*n* = 352 LGBT+ **Sex**: Female: 40.3% Male: 58.5% Trans: 1.2% **Age:** M (SD) 34.46 (12.38) **Sexual Orientation** Gay: 55.1% Lesbian: 27.0% Bisexual: 14.5% Queer: 1.7% Pansexual: 1.7%	CFA	1 and 2 Factors	Estimator: ML with S-B Adjustments	A/C∼∼Som: 0.89	∝ = 0.89	NR
8.	Saldivia et al. (31) Chile	*n* = 1,738 **Sex**: Female: 40.3% Male: 58.5% Trans: 1.2% **Age:** M (SD) 34.46 (12.38)	CFA	1 Factor	Estimator: WLSMV	NR	∝ = 0.89 ω = 0.90	NR
9.	Smith et al. (32) Perú	*n* = 1,098 Pregnant women **Age**: M (SD): 28.1 (6.3)	CFA	1 and 2 Factors Somatic and Affective Symptoms	Estimator: MLR	Som∼∼Affect: 0.82	∝ = 0.80	NR
10.	Aslan et al. (33) Chile	*n* = 1,098 **Gender**: Women: 65% Men: 35% **Age:** 65–80+ 64–69: 36% 70–79: 61% 80+: 4%	CFA	1 and 2 Factors	Estimator: WLSMV	Som∼∼Affect: 0.973	∝ = 0.78 ω = 0.79	NR
11.	Huarcaya-Victoria et al. (14) Perú	*n* = 200 Medicine students **Gender**: Women: 63.5% Men: 36.5% **Age:** M (SD) 20.84 (3.14)	EFA and CFA	1, 2, and Bifactor	Estimator: PC and ML Rotation: Varimax	Som∼∼Affect: 0.87 and 0.88	∝ = 0.903	NR
12.	Quiñones-Freire et al. (34) Ecuador	*n* = 366 Patients of public health care system **Sex**: Female: 65.3% Male: 34.7% **Age:** M (SD) 32.91 (10.56)	CFA	1 and 2 Factors	Estimator: WLSMV	Som∼∼Affect: 0.93	∝ = 0.852 ω = 0.855	Sex
13.	López-Guerra et al. (35) Ecuador	*n* = 5,394 College students **Sex**: Female: 54.8% **Age:** 17–58 M (SD): 22.03 (3.05)	CFA	1, 2, and 3 Factors	Estimator: ML	NR	ω = 0.90 Som: ω = 0.81 Cog/Af: ω = 0.87 Con/Mot: ω = 0.69	Gender and Age

NR, not reported.

Regarding the factorial design method, only three studies initially started with exploratory factor analysis [EFA; (14, 25, 27)] and then moved on to use confirmatory factor analysis (CFA). In all studies examined, they used CFA. Only two implemented a bifactor model technique (14, 35); however, in the study of Huarcaya-Victoria et al. (14), they allowed the two specific factors to be correlated, which is not what is usual in bifactor modeling because a bifactor model is supposed to be uncorrelated (36, 37). In both studies, the interpretation of the specific factors seemed irrelevant, because only the general score was interpreted. However, Huarcaya-Victoria et al. (14) did not use ancillary statistics (i.e., PCU, ECV, ARPB, ω_*H*_, ω_*HS*_) to better analyze the bifactor model (36, 37). This problem was also found in research conducted outside of Latin America [e.g., (38)]; it appears that this issue with the interpretation of the bifactor model is not specific to the context of Hispanic studies of PHQ-9. Only López-Guerra et al. (35) implemented these ancillary statistics beyond the global adjustment, to evaluate the strength of the general factor more accurately and of the items (36, 37).

Concerning the estimator used in CFA analyses, five studies used the “WLSMV” (26, 29, 31, 33, 34), four studies used “maximum likelihood” [ML; (14, 25, 28, 35)], one study used “robust maximum likelihood” [MLR; (32)], one study used the ML estimator with the Satorra-Bentler adjustments (24), and one study did not report the estimator used (27). The results of this brief systematic review are unexpected in terms of estimators because there is literature that claims, for instance, that the WLSMV estimator performs better when there are inter-item polychoric correlations when items are treated as categorical variables [e.g., (39, 40)].

In terms of the internal structure of PHQ-9, two of the studies examined only a one-factor model (24, 31), and two studies examined only a two-factor model (25, 28). Meanwhile, seven studies examined one- and two-factor models (14, 26, 29, 30, 32–34). Only one study examined one-, two-, and three-factor models (35) and only one study examined two- and three-factor models (27). In terms of the conclusions about the internal structure of the PHQ-9 Spanish version, seven concluded that the unidimensional model is the best or at least the more parsimonious explanation of its scores (24, 26, 29–31, 33, 34). On the other hand, three studies concluded that the two-factor model was the best fitted (25, 28, 32) even though the correlation between the two specific factors was very high (*r* = 0.91; (28)). Only one study supported a three-factor internal structure of PHQ-9 (Marcos-Nájera et al. (27)), which they named cognitive/affective symptoms, pregnancy symptoms, and somatic symptoms. Finally, two studies (14, 35) supported the presence of a dominant general factor but also suggested the existence of distinct subcomponents (somatic, cognitive/affective, and concentration/motor).

This discrepancy in classifying the PHQ-9 as multidimensional or unidimensional may result from, among other things, the use of various methods and criteria to determine the PHQ-9′s number of dimensions. However, multidimensionality can be a consequence of response tendencies irrelevant to the content of the instrument, produced by careless response, acquiescence, etc. (41, 42). This variability can be operationalized as a factor in structural equation modeling alone, alongside other types of method variability, or in combination. The multidimensionality discovered may be partially explained by this potential source of method variability, which has been supported by studies examining other measures [e.g., (42)]. However, this source of method variability was not examined in any of the prior validation studies of PHQ-9. Regarding measurement invariance, only five studies examined the measurement invariance of the PHQ-9 Spanish version (24, 28, 29, 34, 35) for sex, gender, education, socioeconomic status, marital status, and/or residence area. This suggests that equivalence between groups is one of the least explored properties, even though it is important to assess the differences in the construct measured in general, and the PHQ-9 in particular.

In terms of reliability, all studies reported Cronbach’s alpha, except the one reported by Cassiani-Miranda and Scoppetta (28). Meanwhile, only five studies reported McDonald’s omega (29, 31, 33–35). Thus, all studies examined in this brief literature review reported reliability coefficients that were well above 0.70.

### Research purpose

The PHQ-9 Spanish version has been used in Puerto Rico, but only a study has examined its psychometric properties (30). Moreover, no study in Latin America or Puerto Rico has examined its psychometric properties with individuals in the workplace context. Therefore, the purpose of this study was to examine the internal structure, psychometric properties, and measurement invariance of the PHQ-9 Spanish version with a sample of workers in Puerto Rico.

The internal structure was investigated in depth to help resolve the apparent multidimensionality of the PHQ-9, somewhat in contrast to the use proposed since its creation, which was one-dimensional (16, 43–45). The association with other variables was also investigated, by linking its association with a measure of burnout, since meta-analytic literature has highlighted its covariation and differentiation [e.g., (46)].

## Materials and methods

### Participants

A total of 969 protocols from two different studies conducted by the authors in Puerto Rico were employed (47, 48) and each one was selected through a non-probabilistic sample and distributed into two groups, namely, sample 1 (*n* = 451) and sample 2 (*n* = 518). The characteristics of the whole and individual samples, such as gender and age, are presented in Table 2. The sample was composed of 55.4% of women and the average education was 15.07 ±2.79, which is equivalent to 3 years of undergraduate studies.

**TABLE 2:** Sociodemographic characteristics of the sample.

Variable	Total sample (*n* = 969)		Sample study 1 (*n* = 451)		Sample study 2 (*n* = 518)	
	***n***	**%**	***n***	**%**	***n***	**%**
**Gender**						
Male	382	39.4	195	43.2	187	36.1
Female	537	55.4	248	55.0	289	55.8
Age (in years)						
21–30	288	29.7	144	31.9	144	27.8
31–50	500	51.6	241	53.4	259	50.0
≥51	181	18.7	66	14.6	115	22.2
**Time working (in years)**						
1–5	278	28.7	130	28.8	148	28.6
6–10	160	16.5	98	21.7	62	12.0
11–15	164	16.9	74	16.4	90	17.4
16–20	124	12.8	51	11.3	73	14.1
21–25	106	10.9	44	9.8	62	12.0
26–30	75	7.7	31	6.9	44	8.5
31	50	5.2	22	4.9	28	5.4
**Job position**						
Managerial	191	19.7	99	22.0	92	17.8
Non-Managerial	750	77.4	348	77.2	402	77.6
**Employment type**						
Tenure	750	77.4	356	78.9	394	76.1
Contract	202	20.8	93	20.6	109	21.0
**Organization type**						
Public	318	32.8	143	31.7	175	33.8
Private	632	65.2	307	68.1	325	62.7
	M	SD	M	SD	M	SD
Education	15.07	2.79	14.30	3.20	15.82	2.06

*n* = 1,829.

### Measures

#### Depression

The PHQ-9, developed by Kroenke et al. (16), was used to assess depression. The PHQ-9 is a nine-item questionnaire used in primary care settings to detect depressed symptoms. This questionnaire assesses the existence of depressive symptoms in the two weeks preceding the completion of the test. Each item is graded on a scale of 0 (not at all) to 3 (very) (nearly every day). Its diagnostic validity and reliability, as well as its utility in assessing depression severity and monitoring treatment response, have all been established (16, 43–45).

#### Burnout

To assess burnout, we utilized the Maslach Burnout Inventory - General Scale [MBI-GS; (49)]. The MBI uses a seven-point frequency scale (range from 0-daily) to indicate how frequently they encountered each item. Emotional exhaustion and cynicism each have five items, whereas professional efficacy has six. In this study, we used the ULSMV estimator to test a three-dimension model, with χ^2^ = 659.871 (87), Z = 22.617, *p* < 0.001, CFI = 0.880, uSRMR = 0.068 [90% CI, 0.067–0.070], and RMSEA = 0.131 [0.121–0.140] without item 13 because we obtained a low, negative, and non-significant factor loading (λ = −0.091) and some studies in Latin America have suggested that some items of MBI-GS are problematic [e.g., (50, 51)]. Specifically, item 13 has been shown to have some factorial complexity in a study conducted in Puerto Rico with a sample of Puerto Rican employees (52). Meanwhile, reliability was estimated for the three subscales, i.e., exhaustion, cynicism, and professional efficacy, using alpha and omega with their respective 90% confidence intervals. Emotional exhaustion obtaining ∝ = 0.912 (90% CI, 0.892–0.929) and ω = 0.910 (90% CI, 0.888–0.928), cynicism ∝ = 0.736 (90% CI, 0.685–0.777) and ω = 0.755 (90% CI, 0.698–0.799), and professional efficacy, ∝ = 0.914 (90% CI, 0.884–0.936) and ω = 0.912 (90% CI, 0.885–0.935). An item example is “I feel tired when I get up in the morning and have to face another day on the job.”

#### Social desirability

The Social Desirability Scale was developed by Rosario-Hernández and Rovira Millán (53). This is an 11-item instrument with a Likert-agreement answer format that ranges from 1 (Totally Disagree) to 6 (Totally Agree), ostensibly measuring a response bias in which people respond to a test by thinking about what is socially acceptable. The internal consistency, as measured by Cronbach’s alpha, is 0.86, which is an outstanding reliability coefficient, according to the authors. The Social Desirability Scale’s internal structure appears to have only one dimension, according to factor analysis results. We used the ULSMV estimator to examine the internal structure of the Social Desirability Scale, and the results support a one-factor structure as reported by the authors: χ^2^ = 531.286 (44), Z = 5.619 (*p* < 0.001), CFI = 0.926, uSRMR = 0.053 [90% CI, 0.052–0.054], and RMSEA = 0.169 [90% CI, 0.157–0.171. Meanwhile, reliability using alpha and omega was 0.930 (95% CI, 0.913–0.943) and 0.928 (95% CI, 0.912–0.942), respectively. An item example is “Most people have cheated on an exam, even if it was once in their lives.”

### Procedures

The first part of the analysis consisted of identifying and removing insufficient effort/careless responses (IE/C). For this, the D^2^ distance (54) was used, a method to detect inconsistent response patterns and expressed as multivariate outliers (55). The R careless program was used (56).

In the second part, and as a content validity source (57), an item analysis was carried out using descriptive statistics to determine the response trend, and the construct validity of the items, through their associations with demographic variables, such as sex, age, education, and the construct of social desirability. The Glass rank biserial, Eta squared, and Spearman rho (58) coefficients were used with the R package rcompanion (59).

In the third part, the PHQ-9 measurement model was evaluated by confirmatory factor analysis within the SEM framework. Several models were fitted to the data: the first model was the congeneric unidimensionality of the items, which is the model usually evaluated in the literature. The second one was a two-factor correlated model, in which items 1, 2, 6, 7, 8, and 9 load on a cognitive/affective content factor, and items 3, 4, and 5 define the somatic content. This model has been reported in several studies (14, 27, 28, 33–35), where both factors were interpreted substantively. The third model was the bifactor, which consists of a general factor and specific factors; this general factor represents the total or global dimension of the content of the PHQ-9, and the specific factors represented the contents of the two-factor model, that is, factor 1(cognitive/affective symptoms) and factor 2 (somatic symptoms). The bifactor modeling allowed us to decide with more information on the multidimensionality or unidimensionality of the PHQ-9, by evaluating the strength of the general factor and the specific factors (37). The final model was a random intercept factor [random intercepts factor analysis: RIFA; (41)], consisting of a substantive factor and a method factor. This method factor estimates individual differences in response scale use and is sensitive to a range of possible causes of response patterns (42). The Steenkamp and Maydeu-Olivares (42) proposal was used, in which each item was freely estimated in the substantive factor but starting with values of one in every item in the random intercepts factor (RIF). This model was included to represent a form of multidimensionality that can compete with the model of two correlated factors. The RIF model was not explored in any of the studies reviewed. The third model was the tau-equivalent model, where the items were constrained to be equal in their factor loadings, something expected for the correct estimation of the alpha coefficient (60).

To examine whether the second (somatic) factor of the two-factor model is a substantive factor or whether it is one that can be attributed to method effects, it was decided to randomly assign five sets of three items to the somatic factor and the remaining items to the cognitive/affective factor. The fit indices of the two-factor model with the different sets of items were then examined. As part of the strategy to randomly assign the items, it was established that only one item of the three somatic factors (3, 4, 5) could be included in case one of them was selected in the randomization process.

To obtain the robust standard error and perform the exact fit test, the ULSMV [unweighted least squares mean-and-variance-adjusted; (40)] estimator was used on inter-item polychoric correlations This method tends to give more accuracy in the estimated parameters (61), particularly with ULSMV due to its sensitivity to detect poorly specified models (62–65). In assessing the fit of the models, the exact fit χ^2^-test (66), residuals, and approximate fit indices (CFI > 0.95; RMSEA ≤ 0.05) were observed. However, the SRMR (the average of the absolute value of residual correlations) was used as the preferred method to evaluate the exact fit, since it is relatively free of the estimation method (67) and accurate for ordinal data (68, 69) using the unbiased estimation of its confidence interval and hypothesis test (68, 70). The two-index strategy was used (64), in which the adjustment was established with the maximum standardized residual covariance (r_res–max_) between a pair of items and the SRMR was adjusted for the size of the factor loadings; fit was close fit (r_res–max_ ≤ 0.10 and SRMR ≤ 0.031; for acceptable fit, maximal r_res–max_ ≤ 0.15 and SRMR ≤ 0.063. Misfit was also observed by correlations of residual ions between the items (71); attention was paid to residual correlations at three levels, i.e., 0.10, 0.20, and 0.30 (64).

Measurement invariance (MI) was studied by sex groups, using a multigroup confirmatory factor analysis [MGCFA; (72)]. A hierarchical evaluation of statistical tests of equivalence was made, starting with the equality of the number of dimensions (configural invariance), factor loads and thresholds (metric invariance), intercepts (scalar invariance), and residuals (residual invariance). Approximate fit indices (AFI: CFI, SRMR, and RMSEA) were also used to estimate the amount of mismatch between one MI model and another. The fit criteria in each measurement invariance model used the recommendations of Chen (73) for a size of 300 participants in each compared group: for metric invariance (SRMR < 0.030, CFI ≤| −0.010|, RMSEA ≤0.015) and scalar invariance (SRMR < 0.015, CFI ≤| −0.010|, RMSEA ≤0.015).

The statistical test of invariance and the AFI were supplemented with indicators of impact or magnitude of non-invariance, specifically dMACS (74). This estimates the impact of between-group differences in the factor loadings and intercepts on the standardized mean difference in the construct (75). This was done by first estimating the configural measurement model independently in the compared groups, under an evaluation of multigroup configural invariance (equality of the number of factors between the groups). Based on the fit to this multigroup model, several interpretable indices were estimated as effect sizes of the degree of non-invariance. At the item level, dMACS is the proportion of the mean difference in observed scores due to differences in intercepts and factor loadings (but not due to differences in the latent attribute). This indicator of the amount of impact attributable to the difference in factorial intercepts and loading is interpreted as <0.20, <0.40, and >0.70 to suggest trivial, moderate, and large non-invariance (76). Another indicator at the item level is the bias in the item mean, centered on the bias that occurred in the responses to each item (item-dMACS). The previous interpretation was used (76). Finally, at the scale level (or total score), the difference in the latent mean was estimated, interpretable as a total or summary indicator of impact or bias, and comparable with the standardized difference Cohen’s *d* (74, 76).

## Results

### Potential insufficient effort/careless responses

With the cutoff point at χ^2^ = 21.66 (df = 9), 114 cases (11.8%) with D^2^ between 21.66 and 90.6 were identified. These participants were removed, and the effective sample for the study was 855.

### Item analysis

The response frequency was asymmetrically distributed, where the options with the highest response frequency were the first two, while option 4 showed low prevalence in items 1, 2, and from 5 to 9. On the contrary, the summary descriptive statistics indicate high mean response similarity (MaxM/MinM = 1.66), focusing homogeneously on response option 1. Items oriented toward somatic symptoms (items 3, 4, 5) tended to show slightly higher mean responses (M = 1.56) compared with the rest of the items (M = 1.23). The distributions were all skewed and positive kurtosis (>1.0), indicating the highest response density in the options referring to fewer depressive symptoms. Inter-item kurtosis was more variable (Max_Ku_/Min_Ku_ = 49.6) than skewness (Max_Sk_/Min_Sk_ = 5.75).

The effect of sociodemographic variables was very close to zero in gender (M = 0.02), age (M = −0.00), and education (M = −0.03). The sample group (M = −0.01) and the social desirability (M = −0.00) were also in this magnitude around zero, which for practical purposes can be considered zero.

### Internal structure

The unidimensional (congeneric) model, where they were not constrained to maintain the same factor loadings, showed a moderately acceptable fit, since the exact fit tests were statistically significant: ULSMV- χ^2^ = 149.494 (df = 27), *p* < 0.0; and uSRMR Z = 2.332, *p* = 0.010. Approximate fit indices indicate a moderate fit (see Table 4, heading Fcong). Possible modifications were examined in this model, and 35 modification indices were obtained (M = 6.55, Md = 3.91; SD = 12.16; max = 72.32 between items 1 and 2). The standardized residuals (M = −0.57) varied between −0.572 (items 2 and 8), and 8.83 (items 1 and 2). Due to the strength of the modification index in the residual covariation between items 1 and 2, this re-specification was introduced but no other re-specifications were added to avoid capitalizing on sampling variability. The re-specified model (unidimensional with modification, e1–e2, in Table 3) with this added residual covariation was satisfactory: ULSMV- χ^2^ = 77.322 (df = 26), p > 0.10; uSRMR Z = 0.855 and *p* = 0.196. However, uSRMR was moderate because its confidence interval was not completely below 0.05 (see Table 4, heading Fmod).

**TABLE 3:** Item univariate analysis (*n* = 855).

Item	Frequencies of response options (PHQ-9)					Descriptive statistics			
	**“Not at all”**	**“Several days”**	**“More than half the days”**		**“Nearly every day”**	**M**	**SD**	**Sk**	**Ku**
PHQ9.1	571	242	39		3	1.38	0.59	1.36	1.33
PHQ9.2	597	223	30		5	1.34	0.57	1.61	2.48
PHQ9.3	528	241	65		21	1.50	0.74	1.43	1.57
PHQ9.4	353	397	86		19	1.73	0.72	0.80	0.43
PHQ9.5	542	232	72		9	1.47	0.69	1.33	1.06
PHQ9.6	729	106	19		1	1.17	0.44	2.67	7.14
PHQ9.7	653	175	26		1	1.26	0.51	1.81	2.71
PHQ9.8	700	134	21		0	1.20	0.46	2.17	4.02
PHQ9.9	816	38	1		0	1.04	0.21	4.62	21.33
**Association with external variables**									
	**PHQ9.1**	**PHQ9.2**	**PHQ9.3**	**PHQ9.4**	**PHQ9.5**	**PHQ9.6**	**PHQ9.7**	**PHQ9.8**	**PHQ9.9**
Sex^a^ (*n* = 810)	0.02	0.02	0.04	0.07	0.02	0.00	0.01	0.04	0.01
Age^b^ (*n* = 805)	−0.03	0.01	−0.05	0.00	−0.04	0.04	0.01	0.04	0.00
Education^c^ (*n* = 804)	−0.04	−0.09	0.01	−0.02	−0.01	−0.07	−0.06	0.00	−0.03
Social desirability^c^ (*n* = 387)	0.0	0.03	0.03	0.02	0.00	−0.04	−0.01	−0.04	0.00
Sample^a^ (*n* = 855)	−0.10	−0.03	0.02	0.03	0.04	−0.03	−0.05	−0.03	0.00

M, mean; SD, standard deviation; Sk, skewness; Ku, kurtosis. ^a^The Glass rank biserial coefficient; ^b^Eta squared coefficient; ^c^Spearman rho coefficient.

**TABLE 4:** Fit indices, factor loadings, and ancillary bifactor statistics of the PHQ-9 models.

Model	Unidimensional		Two-Factor - CFA		Bifactor - CFA					Random intercept factor	
	**Fcong**	**Fmod**	**Cog/Affec**	**Somatic**	**GF**	**SF Cog/Affec**	**SF Somatic**	**I-ECV**	**I-ARPB**	**F**	**Fmet**
PHQ9.1	0.775	0.827	0.711		0.447	0.657		0.316	0.583	0.695	0.189
PHQ9.2	0.899	0.756	0.878		0.542	0.856		0.286	0.599	0.843	0.221
PHQ9.3	0.743	0.786		0.800	0.606		0.487	0.608	0.214	0.709	0.240
PHQ9.4	0.770	0.817		0.783	0.481		0.712	0.313	0.494	0.677	0.529
PHQ9.5	0.805	0.860		0.871	0.643		0.558	0.570	0.241	0.757	0.306
PHQ9.6	0.840	0.781	0.860		0.725	0.450		0.722	0.168	0.852	−0.009
PHQ9.7	0.767	0.826	0.798		0.777	0.263		0.897	0.012	0.812	−0.096
PHQ9.8	0.814	0.746	0.848		0.907	0.180		0.962	0.077	0.856	−0.077
PHQ9.9	0.735	0.827	0.737		0.774	0.169		0.955	0.062	0.800	−0.317
Var	0.600	0.446	0.514	0.640	0.200	0.431	0.237			0.036	
Covariance			0.855								
CFI	0.989	0.995	0.974				0.997			0.995	
RMSEA	0.073	0.048	0.058				0.025			0.057	
Lower	0.062	0.036	0.046				0.000			0.043	
Upper	0.085	0.061	0.070				0.043			0.071	
uSRMR	0.068	0.057	0.044				0.016			0.040	
Lower	0.055	0.043	0.039				0.004			0.032	
Upper	0.080	0.071	0.048				0.027			0.048	
PCU					0.500						
ECV					0.614						
ARPB					0.272						
ω_*H*_					0.742						
ω_*HS*_						0.260	0.440				

Fcong, one-dimensional congeneric model; Fmod, modified model with residual covariation between items 1 and 2; Fri, model with random intercept factor; Fmet, method factor added to Fri; uSRMR, Unbiased SRMR; PCU, percentage of uncontaminated correlation; ECV, explained common variance; ω_*H*_, omega hierarchical; ω_*HS*_, omega hierarchical subscale.

In the exploration of the other substantive models, the correlated two-factor model showed an acceptable fit, χ^2^ = 101.040 (df = 26), *p* < 0.05; uSRMR, Z = −0.286 and *p* = 0.612. A problem detected was the high inter-factorial correlation (*r* = 0.85, *p* < 0.01). In a sensitivity analysis of the adjustment and the high inter-factorial correlation, the adjustment and covariation of this model were compared with a group of two-factor models where the items were distributed at random in two correlated factors to assess the conceptual significance of interpreting two content factors (with the same structure of 6 items in one factor, and 3 items in the other factor). The same number of items dispersed across two separate parameters were extracted from five sets of random items (see Table 5). These five sets all had acceptable levels of fit (uSRMR Z 1.70) and similar levels (CFI 0.950; RMSEA 0.73; uSRMR 0.55). However, there was a very high association between the two factors, and the covariation was between 0.863 and 1.112. The seemingly important two-factor model was not acknowledged as a competitive alternative because of the high repeatable correlation in some combinations of items divided into two components.

**TABLE 5:** Sensitivity analysis—fit indices of different random sets of items for specific factor 2.

Model SF-2 items	χ^2^ (df)	Z (*P*-value)	uSRMR (90% CI)	RMSEA (90% CI)	CFI	Unstandardized covariation
7, 8, 9	109.025* (26)	−1.710 (p = 0.956)	0.044 (0.039–0.050)	0.061 (0.050–0.073)	0.971	0.863
2, 5, 8	128.725* (26)	0.534 (p = 0.297)	0.051 (0.047–0.056)	0.068 (0.057–0.080)	0.964	1.082
1, 3, 6	141..939* (26)	1.692 (p = 0.045)	0.054 (0.050–0.058)	0.072 (0.061–0.084)	0.959	1.026
4, 6, 9	124.347* (26)	0.067 (p = .473)	0.050 (0.045–0.055)	0.067 (0.055–0.079)	0.965	1.112
2, 5, 7	134.162* (26)	0.991 (p = 0.161)	0.053 (0.048–0.057)	0.070 (0.058–0.082)	0.962	1.065

df, degree of freedom; uSRMR, unbiased SRMR; Cov, covariance; Z, Z value based on uSRMR.

*p < 0.05.

A good fit was found for the bifactor model [χ^2^ = 25.798 (df = 17), *p* = 0.078; uSRMR, Z = −5.04, and *p* = 1.00]. However, given that bifactor models tend to outperform conventional CFA models due to the way they are specified (77, 78), it is highly recommended to use complementary statistical indices for a more accurate interpretation when a bifactor model yields adequate model fit indices. Thus, the omega hierarchical for the general factor was ω_*H*_ = 0.742; meanwhile, the omega hierarchical subscale for the cognitive/affective and somatic specific factors was ω_*HS*_ = 0.260 and ω_*HS*_ = 0.440, respectively.

In the general indicators of the model, the explained common variance (ECV) of the general factor did not (0.614) comply with total omega H > 0.80, and this suggests that the total score does not have sufficient psychometric strength. In this line, omega H was less than 0.65, a value that can be considered to be moderate. The average relative parameter bias (ARPB) exceeded the limit of 15%, a value that further suggests a lack of equivalence in the factor loadings of a one-dimensional model and a two-factor model. At this point, there are questions about the strength of a general factor to interpret as a total score. However, the psychometric strength of the specific factors is also not strong enough, and at least one of the specific factors contains variance similarly strong to the general factor. For example, after controlling for the variance of the general factor, the factors do not show acceptable omega H values (cognitive/affective and somatic factors below 0.40). The ECV of the specific cognitive/affective factor seems to be subsumed to the general factor, and while the specific Somatic factor shows more differentiated variance. In other words, the specific Somatic factor contains less variance than the general factor but is not seriously distant from the ECV amount of the specific cognitive/affective factor, and it is moderately independent of the general factor.

At the item level, I-ARPB (Table 4) does not indicate a homogeneous representation of the items for a general factor, because 5 items showed bias greater than 0.20, while in the rest I-ARPB varied between 0.01 and 0.24 (less bias). On the contrary, the I-ECV varied between 0.28 and 0.60 (relatively low variance in the general factor) in the items with the highest I-ARPB, while the other items varied between 0.72 and 0.96. Therefore, although the bifactor model obtained the best approximate fit indices, the complementary indicators suggest a general factor with insufficient strength and inconsistent strength in the specific factors. This inconsistent strength points to the comparatively greater content-specific variance of somatic symptom items, while items with affective/cognitive content show internal inconsistency in representing a specific factor and the general factor. Therefore, given the insufficient strength of both factors, general and specific, the second-best model was accepted: one-dimensional with IR.

Finally, the model with random intercepts (Fri) was comparatively more satisfactory with respect to uSRMR (<0.05) and similar in the other approximate indices, while the exact fit test was also acceptable Z uSRMR = −2.06 and *p* = 0.98 (ULSMV-x2 = 71.79, df = 42, and *p* < 0.01). The difference between the two best models (modified congeneric with residual covariance and random intercepts) was not statistically significant: χ^2^ = 4.47, df = 7, and *p* = 72. Because the RI model can capture individual differences in all items (not only in items 1 and 2), the comparatively best fit but the one-dimensional model with the method factor was also accepted. The variance explained by the method factor can be considered small (variance = 0.036). The Fri model, at this point, was the one that can best represent the variability of the PHQ-9 items.

Additionally, another model tested was the tau-equivalent model; nevertheless, this model was not acceptable: ULSM-x2 = 266.55, df = 35, *p* < 0.01; uSRMR Z = 2.88, and *p* = 0.002; CFI = 0.980, RMSEA = 0.088 (90% CI = 0.078, 0.098), and uSRMR = 0.076 (90% CI = 0.061, 0.091). We intended to examine whether the specific factor 2, which has been called somatic symptoms, obtained good fit indices regardless of the set of items it had. In this way, we randomly selected three items and made up that the specific factor and the rest of the items formed the specific factor 1; as can be seen in Table 4, these five randomly selected sets of items obtained acceptable fit indices.

### Sensitivity analysis

A sensitivity analysis was performed to examine whether the model of two correlated factors obtained acceptable fit indices regardless of the set of items that made up each factor, which were randomly assigned. Table 5 shows that the five sets of randomly assigned items of two-factor correlated models obtained acceptable fit indices.

### Measurement invariance

Due to the low prevalence of response in item 9, it was dichotomized to allow comparison between the groups, and not exclude it from the study’s conclusions. As a reference in the group of men, Table 6 shows that in the evaluation of the metric invariance (i.e., equality of factor loads plus thresholds) the equality of these parameters is not accepted. At the levels of invariance of intercepts and residuals, the differences in AFI were met. From another evaluation angle, the dMACS effect size indicators showed that the differences in factor loadings or intercepts produced a trivial impact (dMACS ≤ 0.20) on most items (dMACS M = 0.13 and dMACS SD = 0. 04). On the contrary, items 3, 5, and 9 did not meet this criterion but are close to it (dMACS ≤ 0.25). This range of values in both groups of items, however, can be considered a small amount of impact (79). The response bias in each item (M = 0.05, SD = 0.03) can be considered trivial, especially in the items that seemed questionable in dMACS (i.e., items 3, 5, and 9). The impact on the total score is 0.464, indicating a moderate effect.

**TABLE 6:** Measurement invariance and effect size (d_MACS_).

	ULSMV-χ^2^ (df)	Approximate fit indices (AFI)			Differences in AFI		
**Model**		**CFI**	**RMSEA (90% CI)**	**SRMR**	**CFI**	**RMSEA**	**SRMR**
Configural	182.251 (54)	0.998	0.077 (0.067, 0.089)	0.070	–	–	–
Metric	285.010 (62)	0.980	0.094 (0.083, 0.106)	0.085	−0.01	0.017	0.015
Scalar	328.176 (69)	0.976	0.096 (0.086, 0.107)	0.086	−0.008	0.002	0.001
Residual	328.176 (78)	0.977	0.089 (0.079, 0.099)	0.086	0.001	−0.007	0.00
**Effect size estimators**							
	**d_MACS_**	**Δ**_mean item_					
PHQ9.1	0.114	0.050					
PHQ9.2	0.207	0.075					
PHQ9.3	0.226	0.077					
PHQ9.4	0.127	0.062					
PHQ9.5	0.251	0.102					
PHQ9.6	0.152	0.035					
PHQ9.7	0.113	0.038					
PHQ9.8	0.086	0.022					
PHQ9.9	0.258	−0.000					

### Association with other variables

To gather and establish the convergent and divergent validity, we correlated the PHQ-9 scores to the three dimensions of the Maslach Burnout Inventory and Social Desirability Scale. We anticipated that the depression scores would significantly positively correlate with emotional exhaustion and cynicism, with moderate to high effect sizes; however, Table 7 shows that these correlations are near zero. On the other hand, we expected low and negative correlations of depression with professional efficacy and social desirability and Table 7 shows that those expectations were met. It is important to mention that we performed the covariation analysis of the latent variables including those 114 cases eliminated due to possible potential insufficient effort and/or careless responses and the magnitudes of these associations vary greatly when cleaning the data; for example, the relationship values of depression with emotional exhaustion and cynicism were 0.713 and 0.678, respectively. These results might suggest an inflation of these relationships due to a methodical effect caused by a lack of proper handling and cleaning of the data.

**TABLE 7:** Association with external variables: Unidimensional and RIF score model.

Scale	Maslach Burnout Inventory – General Survey (MBI-GS)			SD	Sex	Age
	**EE**	**Cyn**	**PE**			
**PHQ-9**						
Unidimensional score	−0.03 (−0.13, 0.06)	−0.04 (−0.14, 0.06)	−0.01 (−0.11, 0.08)	0.04 (−0.05, 0.14)	0.06 (−0.00, 0.13)	−0.14** (−0.21, −0.07)
**PHQ-9 RIF model**						
Unidimensional score	−0.03 (−0.13, 0.06)	−0.04 (−0.14, 0.05)	−0.01 (−0.11, 0.08)	0.03 (−0.06, 0.13)	0.05 (−0.00, 0.12)	−0.14** (−0.21, −0.07)
RIF score	−0.01 (−0.11,.08)	0.03 (−0.06, 0.12)	−001 (−0.11, 0.08)	0.03 (−0.06, 0.13)	0.08* (0.01, 0.14)	−0.06 (−0.12, 0.00)

*p < 0.05; **p < 0.01.

Confidence Interval in 95%, RIF, random intercept factor model; EE, emotional exhaustion; Cyn, cynicism; PE, professional efficacy; SD, social desirability.

## Discussion

The items showed a uniform response pattern with respect to the chosen options; these options were 1, 2, and 3. Only these could be sufficiently informative to describe the symptoms of depression in the general population of workers, in which the prevalence is presumed. Except for the symptoms associated with somatic complaints (sleeping problems, tiredness, and appetite changes), the rest of the items do not usually occur almost every day. The general trend is that the frequency distributions of the items can be considered similar. This response frequency was consistent with summary statistics, where similarity was constant in mean response, spread, skewness, and kurtosis. The exception in this last statistic was item 9 (thought of death or self-harm).

The homogeneity of the descriptive characteristics of the items also occurred in the relationship with other constructs (e.g., social desirability) and with sociodemographic variables. All these correlations were very close to zero, and for practical purposes, they can be considered null correlations. This indicates the independence of the content of the items from the variability originating from these variables. Although it is not a direct measure of differential item functioning, the absence of a relationship indicates an approximation of the absence of trends between the contents of PHQ-9. This is particularly important in relation to social desirability, because it implies that none of the content is affected by this source of systematic variability, when applied as a self-report on workers. This relationship probably increases in an interview where PHQ-9 is included, as has been reported in other contexts and groups of participants (80, 81). Less literature exists on this association in PHQ-9 and in Latin American workers, and we cannot reliably make safe generalization. However, these results imply that, in the context of the face-to-face application and as a self-report, the sampled workers do not bias their responses toward the minimization of depressive symptoms.

Five models were tested in terms of internal structure, starting with models that start with substantive or theoretical interpretation dimensions and ending with models that include a response bias dimension. In this way, models explored in earlier research were reviewed, and eventually a model—the random intercept factor—was incorporated as a hypothetical explanation of multidimensionality. The interpretation of the substantive models typically had issues, which prevented people from evaluating them to be the best model for the data. For example, the two-factor correlated model, with theoretically interpretable factors, showed a high interfactorial correlation, which implies statistical redundancy. In another model, modified unidimensional, the improvement of its fit involved adding a residual covariance to reach the AFI cutoff points; however, this also capitalizes sampling variability, and we only chose the largest residual covariance, which occurred between items 1 and 2. However, this residual covariance does not seem to replicate what was found in the reviewed literature, in which no study mentions the need to covary the residuals of the items to enhance fit indices and, therefore, it may be characteristic of the study sample. Meanwhile, the bifactor model, composed of a general factor and two specific ones with theoretical interpretation, showed a very good statistical fit, but it was not strong with respect to the construction of a general factor. On the other hand, the model with equal factor loadings (tau-equivalent) did not show an acceptable fit and was discarded, implying that the validity of each item cannot be concluded to be equal. Finally, the alternative model proposed in this study, different from other studies, influences the model with random intercepts, which obtained acceptable fit indices. Maydeu-Olivares and Coffman (41) argued that the assumption of common linear coefficients may be overly restrictive when the observed variables are participants’ observed responses to stimuli, such as their responses to the items of a questionnaire. This might happen, for instance, if participants consistently use the response scale in an idiosyncratic way (103). By allowing the intercepts in the factor model to vary across participants, the model partially relaxes the fixed coefficient assumptions to account for this phenomenon.

One implication of these results is that the emergence of an additional factor may be due to response patterns that are irrelevant to the measured construct, and that are expressed by different sources of variability. This possible variability may be partially focused on some PHQ-9 items, and since the studies that support the two-factor model separate the somatic response items from the rest, these items are probably associated with responses with irrelevant trends. The magnitude of the factor loadings of these items in the method factor in RIFA seems to tangentially support this conjecture. But a qualitative exploration is required; for example, a cognitive interview focused on this possible problem (81). The impact of variability on the use of the RIF response scale did not have a serious impact on another source of validity; specifically, in relation to other variables (see below), there were insubstantial differences between the correlations of the external variables with PHQ-9 and with method factor. Therefore, a superior fit of the RIF model does not necessarily imply that it will have a substantial impact on other validity indicators.

Regarding the reliability estimates obtained, the total score of PHQ-9 can yield highly reproducible scores (ω = 0.90), but the presence of potential individual differences in the use of the response options deteriorates reliability. This impairment is below 0.90 but still above 0.80, indicating that PHQ-9 can be optimally used for screening and group descriptions.

In the evaluation of the invariance, all the tested models were satisfactory, but with some caveats to consider. In metric invariance, where factor loadings are constrained to be equal across groups, the AFI indicators highlighted possible non-invariance. However, the evaluation of the impact of this potential invariance was aided by dMACS and its accompanying indicators. On the total scale, a moderate impact difference, but derived from predominantly small biases at the item level, was observed (79). Due to the small amount of impact, the rest of the invariance tests continued. Thus, strict and scalar invariance satisfied the difference criteria in the AFI (73).

As reported in a methodological study (82), with the correct specification of the dimensionality of PHQ-9 (in this study, the accepted unidimensionality without the RIF), and high factor loadings (i.e., the strength of the items to represent the construct) the difference between the invariance results of the factor loadings and the residuals is trivial, and therefore, the strict measurement invariance (i.e., equality of configuration, factor loadings, intercepts, and residuals) can be concluded without directly testing this last level of invariance (82).

In terms of the relationship between the scores of PHQ-9 and the three-dimensional subscales of MBI-GS and social desirability scales, these results were partially supported. Regarding convergent validity, the association between the scores of PHQ-9 and emotional exhaustion and cynicism subscale scores was near zero, which was not expected; however, these results are consistent with some of the studies [e.g., (83–85)]. Moreover, some studies argue that depression and burnout are two separate constructs (86). Although there are some similarities between burnout and depression, such as a lack of energy, several researchers disagree and contend that emotional exhaustion is unrelated to depression (87). In contrast, there is literature that has shown a positive correlation between depression and burnout [e.g., (87–89)]. In fact, as Bianchi and Laurent (88) indicated in their systematic review, it has been found that inventories that assess burnout, and more specifically the subscale of emotional exhaustion, the core component of burnout, are positively correlated with depressive symptoms (90–92). On the contrary, divergent validity was examined *via* the relationships between the scores of PHQ-9 and the Professional Efficacy subscale of the MBI-GS and the Social Desirability Scale were as expected, negative. In the case of the relationship between depression and professional efficacy, our results concord with some studies [e.g., (93)]. Meanwhile, our results are comparable to other studies [e.g., (94)] in which these variables correlated negatively, supporting the divergent validity of the scores of PHQ-9.

Regarding the cultural and clinical implications of the results of this study, it should be considered that the use of PHQ-9 was in the workplace, which will always generate some type of stress and can lead to the manifestation of depression symptoms and other conditions (e.g., anxiety). In addition, we cannot lose sight of the syndrome known as “ataque de nervios,” which has been documented in several studies with Puerto Ricans (95, 96). “Ataques de nervios,” a cultural syndrome common in Puerto Ricans, is defined as a severe emotional outburst brought on by extreme stress. A recent study conducted in Puerto Rico by Roche-Miranda et al. (97) found that people with “ataques de nervios” syndrome showed more depression symptoms than those without the syndrome. Furthermore, there is research in which it was found that “ataques de nervios” is also manifested in Latinos living in the USA, which includes Puerto Ricans, Mexicans, Cubans, among others [e.g., (98)]. Another factor to be considered is that women tend to report more “ataques de nervios” than men (96). This is particularly important in the case of Puerto Rican working women who, according to their cultural traditions, must also fulfill their socially anticipated duties as mothers, spouses, and caregivers. To improve our clinical practice as culturally competent healthcare professionals, we agree with Roche-Miranda et al. (in press) that it is crucial to consider cultural aspects when providing clinical tools to understand the population of employees as patients.

### Limitations and recommendations

The current study has several limitations that need to be pointed out. First, population representativeness is not guaranteed, because the non-random selection of the samples did not corroborate the population similarity. Thus, it is necessary to continue investigating the scores produced by PHQ-9 in the context of employees in Puerto Rico. Second, when the fit of the two-factor model solutions with randomly drawn items was verified experimentally, sensitivity analyzes were not performed with a larger number of random samples of the items (for example, 100 or more); therefore, the estimate obtained has a large variability of sampling. For this reason, it is recommended to replicate this type of study by increasing the random samples of the items. Third, another limitation concerns the examination of measurement invariance because there is no consensus on the application of criteria for measurement invariance within the SEM approach, since they do not have a robust quality for their application in different situations faced by the researcher (99). Therefore, different criteria may indicate different results, with no knowledge of Type I or Type II errors occurring. Permutation approaches may be required (100) that achieve better control rates for Type I errors (100, 101). It is possible that item 9, which was dichotomized due to its low prevalence of response in one of the groups, requires modeling PHQ-9 with a model that considers this. Finally, regarding the relationship between depression and burnout, emotional exhaustion and cynicism in the current study were near zero when eliminating cases that showed insufficient effort and careless responses; however, when the whole sample was used, the relationship between these constructs was inflated. Therefore, it is necessary to examine these methodical effects on the relationship of these constructs in the future, for example, effects on the sensitivity of fit indices due to faking in questionnaires [e.g., (102)]. Another possible factor that affected the relationship between depression and burnout could be related to the poor fit of the MBI-GS in this sample. Thus, this is another factor that needs to be examined in the future despite the fact that a study (52) with a sample of Puerto Rican workers obtained excellent fit indices although they used the exploratory structural equation modeling (ESEM) in their study, which allows cross-loadings and the fit indices tend to become better.

## Conclusion

The current study intended to examine, in-depth, the internal structure to help resolve the apparent multidimensionality of PHQ-9. Several models were tested; for example, the unidimensional model was acceptable, but it was needed to covariate error terms of items 1 and 2. The two-correlated factors show an acceptable fit, but the interfactorial correlation was too high. The bifactor model was the best-fitted one; however, it was not strong with respect to the construction of a general factor. The alternative model proposed in this study was the random intercepts that obtained acceptable fit indices. Moreover, a sensitivity analysis was conducted to examine the two correlated factor models regardless of the set of items that made up each factor randomly assigned, and the five sets obtained acceptable fit indices; therefore, this result suggests that the somatic factor of the two correlated factor model is not a substantive factor after all. Regarding the interpretation of the PHQ-9′ scores, it can be said that, for now, a one-dimensional structure is the most parsimonious interpretation, as some researchers have argued (24, 26, 29–31, 33, 34) until more studies are carried out that examine response patterns or variability due to method effects.

## Data availability statement

The original contributions presented in this study are included in the article/supplementary material, further inquiries can be directed to the corresponding author.

## Ethics statement

The studies involving human participants were reviewed and approved by Ponce Health Sciences University. The patients/participants provided their written informed consent to participate in this study.

## Author contributions

CM-S and ER-H: formal analysis and supervision. ER-H and LR-M: investigation. ER-H: funding acquisition. All authors conceptualization, methodology, writing—original draft preparation, and writing, reviewing, and editing.
